# Comparison of endoscopic bilateral areolar and robotic-assisted bilateral axillo-breast approach thyroidectomy in differentiated thyroid carcinoma: a propensity-matched retrospective cohort study

**DOI:** 10.1186/s12893-023-02250-w

**Published:** 2023-11-08

**Authors:** Xiaokang Fu, Yunhan Ma, Yiqi Hou, Yuan Liu, Luming Zheng

**Affiliations:** 1 Jinzhou Medical University, Jinzhou, Liaoning 121000 China; 2 Department of Thyroid and Breast Surgery, the 960th Hospital of People’s Liberation Army, No.25, Shifan Road, Tianqiao District, Jinan, 250031 China

**Keywords:** Differentiated thyroid carcinoma, Bilateral axillo-breast approach robotic-assisted thyroidectomy, Endoscopic bilateral areolar approach, Propensity score-matched

## Abstract

**Background:**

Robot-assisted and endoscopic thyroidectomy are superior to conventional open thyroidectomy in improving cosmetic outcomes and postoperative quality of life. The procedure of these thyroidectomies was similar in terms of surgical view, feasibility, and invasiveness. However, it remains uncertain whether the robotic-assisted bilateral axilla-breast approach (BABA) was superior to the endoscopic bilateral areolar approach (BAA) thyroidectomy. This study aimed to investigate the clinical benefit of these two surgical procedures to evaluate the difference between these two surgical procedures by comparing the pathological and surgical outcomes of endoscopic BAA and robotic-assisted BABA thyroidectomy in differentiated thyroid carcinoma.

**Methods:**

From November 2018 to September 2021, 278 patients with differentiated thyroid carcinoma underwent BABA robot-assisted, and 49 underwent BAA approach endoscopic thyroidectomy. Of these patients, we analyzed 42 and 135 patients of endoscopic and robotic matched pairs using 1:4 propensity score matching and retrospective cohort study methods. These two groups were retrospectively compared by surgical outcomes, clinicopathological characteristics, and postoperative complications.

**Results:**

The mean operation time was significantly longer in the EG than in the RG (*p* < 0.001), The number of retrieved lymph nodes was significantly lower in the ET group than in the RT group (*p* < 0.001). The mean maximum diameter of the thyroid was more expansive in the EG than in the RG (*p* = 0.04). There were no significant differences in the total drainage amount and drain insertion days between the two groups (*p* = 0.241, *p* = 0.316, respectively). Both groups showed that cosmetic satisfaction (*p* = 0.837) and pain score (*p* = 0.077) were similar. There were no significant differences in complication frequencies.

**Conclusion:**

Robotic and endoscopic thyroidectomy are similar minimally invasive thyroid surgeries, each with its advantages, both of which can achieve the expected surgical outcomes.

**Trial registration:**

Retrospectively registered.

**Supplementary Information:**

The online version contains supplementary material available at 10.1186/s12893-023-02250-w.

## Introduction

In recent years, the incidence of thyroid cancer has continued to rise worldwide. Differentiated thyroid cancer (DTC) is the most common subtype of thyroid cancer, and for most patients, surgery is an effective treatment [1–3].

Since Gagner first performed endoscopic surgery on the head and neck in 1996 [4], and the first endoscopic thyroidectomy was performed by Hüscher in 1997 [5], endoscopic thyroidectomy has become widespread over the past few decadesx [5]. With the advancements in high-definition endoscopy and robotic assistance systems, including axillary, breast, anterior chest, postauricular facelift, and transoral routes, the endoscopic remote access thyroidectomy has been developed to minimally scarring for improving the quality of life [6–9]. The remote access approach provides excellent cosmesis and a magnified surgical view [6]. Our previous research and other studies have established the superiority of robotic-assisted thyroidectomy and endoscopic thyroidectomy procedures over conventional open surgery in improving cosmetic outcomes and postoperative quality of life [9, 10].

The bilateral areolar approach (BAA) and bilateral axilla-breast (BABA) approaches have become the most common methods for endoscopic and robotic-assisted thyroidectomy [11]. Both approaches were similar regarding surgical view, feasibility, and invasiveness. Some previous studies compared endoscopic thyroidectomy with robotic thyroidectomy based on the BABA or transaxillary approach [12–14]. However, no study has compared the BAA in endoscopic thyroidectomy (ET) with the BABA in robotic thyroidectomy (RT) in DTC patients. Since endoscopic and robotic-assisted thyroidectomy had comparable postoperative complications and locoregional recurrence rates [14], we try to analyze the differences in surgical outcomes between ET and RT. This study aimed to investigate the clinical benefit of these two surgical procedures to evaluate the difference between these two surgical procedures. We compared the surgical outcomes of endoscopic BAA thyroidectomy versus robot-assisted thyroidectomy using the Bilateral axilla-breast approach in DTC and used propensity score matching to minimize bias in preoperative baseline data.

## Materials and methods

### Patients

**Fig. 1:**
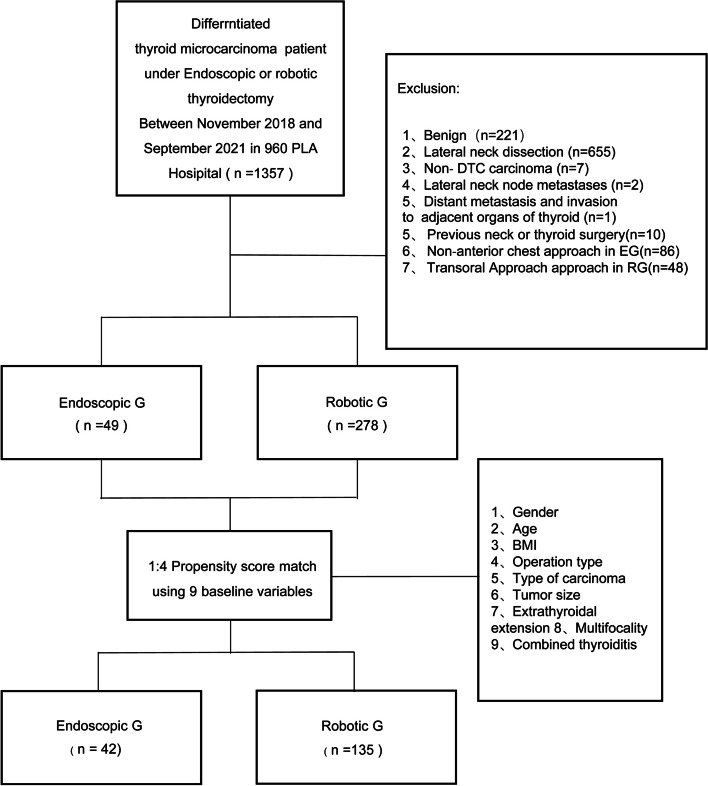
Study flow chart

We retrospectively reviewed 1357 patients who underwent thyroidectomy using endoscopic or robotic-assisted procedures from November 2018 to September 2021 in the 960th PLA Hospital (Ji Nan, China). A total of 1030 patients were excluded based on the following criteria: Benign (*n*=221), lateral neck dissection (*n*=655), Non- DTC carcinoma (*n*=7), Lateral neck node metastases (*n*=2), Distant metastasis and invasion to adjacent organs of the thyroid (*n*=1), Previous neck or thyroid surgery(*n*=10) and Non-anterior chest approach (*n*=86) or transoral approach (*n*=48), we were recommended to evaluate the thyroid gland as well as central and lateral neck lymph node chains using the preoperative neck ultrasonography as the initial step [15]. Then, preoperative laryngoscopy was used for routine assessment of the vocal fold motion, and thyroid function tests were performed to assess thyroid function abnormalities [16, 17]. Following the ATA and NCCN guidelines, the initial surgical procedure should be a thyroid lobectomy unless there are clear indications to remove the contralateral lobe [3, 18, 19]. Therefore, this study included patients who underwent lobectomy or total thyroidectomy with central neck dissection (CND). Through adequate preoperative explanation of BAA in ET and BABA in RT, the operation method was decided according to the patient’s preference. Patients were categorized into two groups according to the type of surgery. The medical charts and pathology reports of 327 patients were reviewed and analyzed using retrospective cohort study methods. After propensity score matching(PSM), we eventually included 177 patients who underwent thyroidectomy with CND for DTC in this study. Forty-two patients were included in the ET group, and 135 patients were included in the RT group. A flow chart of the study is presented in Fig. 1. Ethical approval for this study (Ethical Committee NO.157) was provided by the Ethical Committee of the 960th Hospital of the People’s Liberation Army, Jinan, China on 26 December 2022. Due to the retrospective nature of this study, the Ethical Committee waived the requirement for informed consent.

### Operation methods

In our institutions, three surgeons with > 10 years of experience in thyroid and endocrine surgery were involved in the operation. All thyroidectomies were performed by the same experienced surgeon teams with the da Vinci Si Surgical Robot System (Intuitive Surgical, Inc., Sunnyvale, CA). A neural integrity monitor was utilized for all the surgery [20]. The day before the operation, 0.05-0.1 ml of carbon nanoparticles’ suspension injection was injected into the left and right lobe thyroids under the guidance of ultrasound to leave the parathyroid glands unstained and staining level VI area lymph nodes. Previous studies have demonstrated that robotic and endoscopic procedures are very similar, except for using a robotic arm [12, 13]. The difference is that the BABA approach has more incisions in the axilla than the BAA approach [9]. Therefore, our study’s endoscopic and robotic thyroidectomy surgical procedures were virtually identical. Therapeutic CND when suspicious lymph node enlargement is detected on preoperative or intraoperative examination, prophylactic CND is for patients with clinically uninvolved central neck PTC, especially for advanced primary tumors [18].

### Surgical procedure

The BABA approach was performed in RG. A 1.5 cm incision was made at the internal edge of the right areola for the insertion of the scope of the surgical system, an incision was made at the edge of the left areola and anterior of the right axilla for the placement of an 8 mm trocar as operating approach, an incision was made anterior of the left axilla for the post of a 5 mm trocar as operational approach, and an incision was made anterior of the right areola for the insertion of a 12 mm trocar as a scope. A robotic surgical system was connected to establish the operating space, and the working area was maintained by CO_2_ with a pressure of 5-6 mmHg. The isthmus of the thyroid was cut with a harmonic scalpel, and the side of the lesion was resected. The bilateral recurrent laryngeal nerves (RLN) were protected using intraoperative neuromonitoring (IONM) [20].

The BAA approach was performed in EG. A 12 mm trocar was inserted into the right areola incision to access the endoscope lens, and 8 mm trocars were inserted into the left and right areola incisions to place the operating instruments. The operational space was maintained by inflation with CO2 at a pressure of 5 to 6 mmHg. The harmonic scalpel was used to divide the isthmus of the thyroid. The thyroid on the focal side was resected. At the same time, the RLN was monitored using the IONM, to preserve the parathyroid glands and protect the RLN. The details of the BABA robotic procedure and the BAA endoscopic procedure were described in the previous report [9, 10].

### Surgical outcomes measured

For comparison of pathological characteristics and surgical outcomes between the two groups, we measured the maximum diameter of the thyroid, number of positive lymph nodes metastasis, total operation time, the total amount of drainage, drain insertion, postoperative pain score (VAS), cosmetic satisfaction, postoperative hospitalization stay, number of retrieved lymph nodes, and postoperative complications. Postoperative laboratory data such as serum calcium and parathyroid hormone were also measured and compared. Hypoparathyroidism was defined as a serum parathyroid hormone (PTH) <15.0 pg/ml, and hypocalcemia was defined as serum calcium <2.08 ng/mL or having positive symptoms after thyroidectomy. The size of the thyroid specimen was determined by the longest diameter of the most oversized thyroid in cases of multiple specimens. The operative time was determined from skin incision to closure. The postoperative pain was measured using the visual analog scale (VAS) score (0–10 points) on the postoperative days; The period of hospitalization was defined from the day of surgery to the day of discharge. RLN injury was defined as vocal cord palsy persisting after six months. Cosmetic satisfaction was assessed on a scale from 0 (dissatisfied) to 3 (extremely satisfied).

### Propensity score-matching analysis

**Fig. 2:**
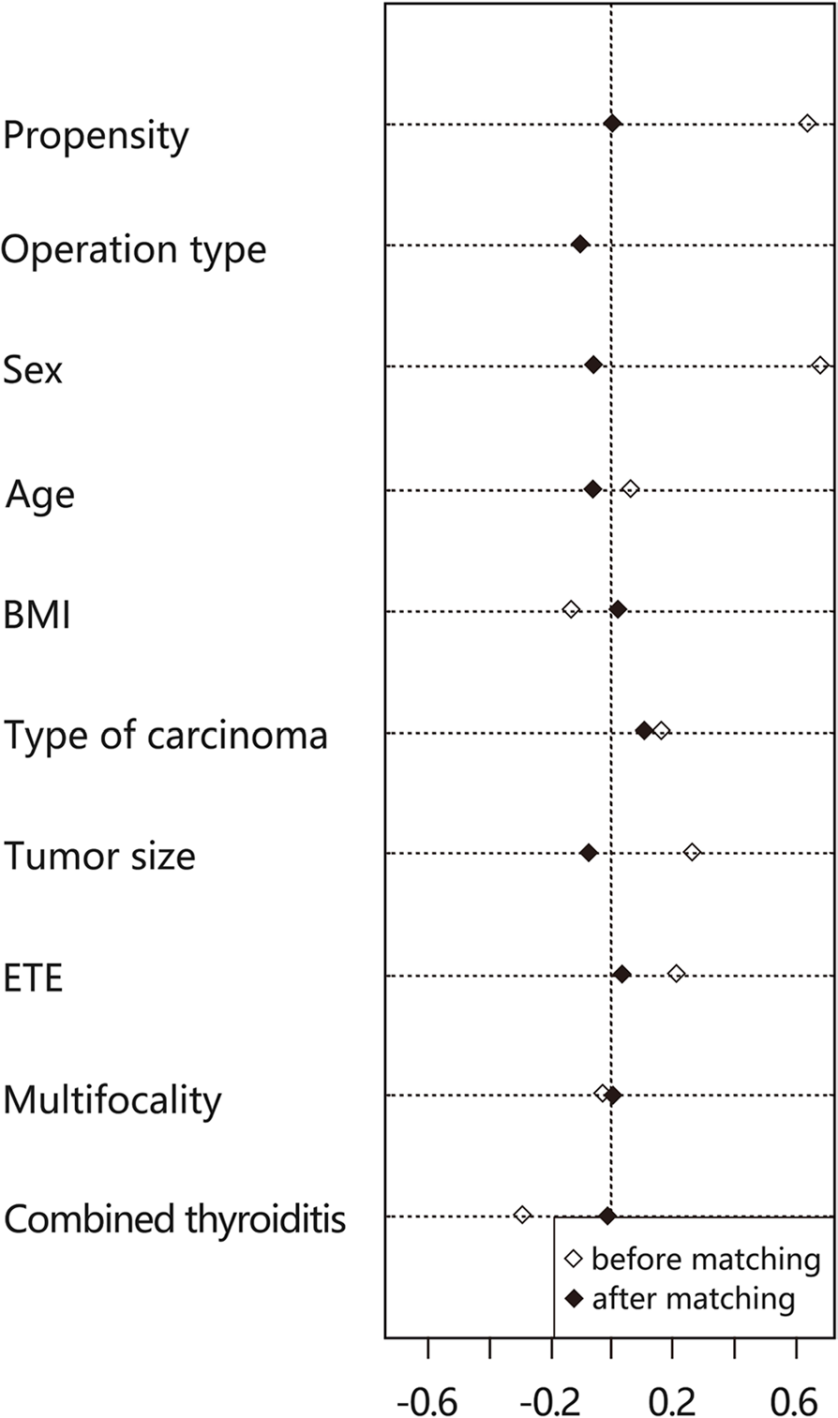
Propensity score matching with 9 variables

To minimize the effects of selection bias, propensity score matching was performed using nine clinicopathological characteristics: age, gender, BMI, tumor size, extrathyroidal extension (ETE), multifocality, the extent of surgery, type of carcinoma, and combined thyroiditis. Individual patient propensity scores were calculated using logistic regression analysis (Fig. 2). Forty-two patients who underwent ET were matched in a 1:4 ratio with 135 patients who underwent RT. Although we presented the TNM stage in Table 1, we did not use the TNM stage as a matching variable for the following reasons. First, the components of the T-stage (tumor size and ETE) were used as matching variables. Then, we initially excluded DTC patients with distant metastasis, including lateral neck node metastases. Finally, retrieved CLN was used as an outcome variable in this study to compare the efficacy of the two groups.

**Table 1:** Clinical and histopathologic characteristics of the patients in the robotic and endoscopic groups before propensity score matching

	**Endoscopic Thyroidectomy (*N*** = **49)**	**Robot-assisted Thyroidectomy (*N*** = **278)**	***P***
Age (yr)	41.3 ± 10.1	40.6 ± 10.3	0.561
Gender			0.002
Male	5 (10.2%)	87 (31.3%)	
Female	44 (89.8%)	191 (68.7%)	
BMI (kg/m2)	24.3 ± 3.5	24.7 ± 3.6	0.586
Operation type			0.466
LT	10 (20.4%)	45 (16.2%)	
TT	39 (79.6%)	233 (83.8%)	
Type of carcinoma			0.048
PTC	47 (95.9%)	276 (99.3%)	
FTC	2 (4.1%)	2 (0.7%)	
Tumor size (mm)	6.55 ± 5.25	5.15 ± 2.67	0.331
Extrathyroidal extension			0.052
Yes	6 (12.2%)	14 (5%)	
No	43 (87.8%)	264 (95%)	
Multiplicity			0.832
Yes	7 (14.3%)	43 (15.5%)	
No	42 (85.7%)	235 (84.5%)	
Tumor location			0.124
Unilateral	41 (84%)	201 (72.3%)	
Bilateral	8 (16%)	61 (21.9%)	
isthmus	0 (0%)	16 (5.8%)	
Combined goiter			0.995
Yes	31 (63.3%)	176 (63.3%)	
No	18 (36.7%)	102 (36.7%)	
Combined thyroiditis			0.118
Yes	7 (14.3%)	68 (24.5%)	
No	42 (85.7%)	210 (75.5%)	
T Category			0.003
1a	37 (75.6%)	252 (90.7%)	
1b	6 (12.2%)	12 (4.3%)	
3b	6 (12.2%)	14 (5.0%)	
N Category			0.534
0	35 (71.4%)	186 (66.9%)	
1a	14 (28.6%)	92 (33.1%)	
Stage			0.907
I	48 (98.0%)	273 (98.2%)	
II	1 (2.0%)	5 (1.8%)	

Values are numbers of patients(percentages) unless otherwise indicated

The underlined values in the table show statistically significant difference between the two groups

*Abbreviations*: *PTC* papillary thyroid carcinoma, *FTC*, Follicular thyroid carcinoma, *LN* lymph node, *TT* total thyroidectomy with CCND, *LT*, Lobectomy with CCND, *BMI* Body Mass Index

### Statistical analysis

Continuous, quantitative data are expressed as means ± standard deviations or median with interquartile range (Q1–Q3), and categorical, qualitative data as frequencies and percentages. The groups were compared using Pearson’s χ2 test, the Mann-Whitney U test, the Student’s t-test, or Fisher’s exact test for qualitative or quantitative variables, as appropriate. The simple linear regression model used surgical times as a dependent variable. The estimates from the regression models were presented corresponding to 95% confidence intervals (CIs). Statistical significance was accepted at the *P* <0.05 level. All the statistical analyses were performed using SPSS 26 (Statistical Package for Social Science, version 23.0; IBM Corporation, Chicago, IL), GraphPad Prism version 9.0 software, and R 3.5.3.

## Results

### Baseline clinicopathological characteristics before and after propensity score matching

The baseline clinical and pathological characteristics of 327 patients before PSM are shown in Table 1. The patients were divided into two groups, including 278 robotic procedures (RG) and 49 endoscopic procedures (EG). There were more male patients in the RG (*p* = 0.002). The patients who performed also had a higher ratio of T1a category, a lower ratio of T1b category, and T3b category in RG (*P*= 0.003). Furthermore, the pathological types differed significantly in the two groups (*P*=0.048). Although the EG had a larger tumor size and more extrathyroidal extension cases than the RG, there were no significant differences found in the two groups (6.55±5.25 mm in EG vs. 5.15±2.67 mm in RG, *p* = 0.331 and 12.2% in EG vs. 5% in RG *p* = 0.052, respectively), The other variables such as age (*p* = 0.561), BMI (*p* = 0.586), multiplicity (*p* = 0.832), tumor location (*p* = 0.124) did not show a statistical difference. The results suggest that the baseline levels of the two groups of data are inconsistent. By using 1:4 propensity score matching, we minimized the bias in baseline data between the two groups. Table 2 shows the two groups’ baseline clinical and pathological characteristics after propensity matching. The EG had 42 patients, and 135 patients underwent RT. There was no significant difference in baseline data between the two groups after propensity matching.

**Table 2:** Clinical and histopathologic characteristics of the patients in the robotic and endoscopic groups after propensity score matching

	**Endoscopic Thyroidectomy (*N* = 42)**	**Robot-assisted Thyroidectomy (*N* = 135)**	***P***
Age (yr)	42.1 ± 9.7	41.6 ± 9.6	0.668
Gender			0.906
Male	5 (11.9%)	17 (12.6%)	
Female	37 (88.1%)	118 (87.4%)	
BMI (kg/m2)	24.3 ± 3.6	24.5 ± 3.9	0.942
Operation type			0.376
LT	9 (21.4%)	21 (15.6%)	
TT	33 (78.6%)	114 (84.4%)	
Type of carcinoma			0.78
PTC	40 (95.2%)	134 (99.3%)	
FTC	2 (4.8%)	1 (0.7%)	
Tumor size (mm)	5.18 ± 3.49	5.32 ± 2.82	0.471
Extrathyroidal extension			0.487
Yes	3 (7.1%)	6 (4.4%)	
No	39 (92.9%)	129 (95.6%)	
Multiplicity			0.842
Yes	6 (14.3%)	21 (15.6%)	
No	36 (85.7%)	114 (84.4%)	
Tumor location			0.256
Unilateral	36 (85.7%)	101 (74.8%)	
Bilateral	6 (14.3%)	30 (22.2%)	
isthmus	0 (0.0%)	4 (2.3%)	
Combined goiter			0.833
Yes	26 (61.9%)	86 (63.7%)	
No	16 (38.1%)	49 (36.3%)	
Combined thyroiditis			0.563
Yes	7 (16.7%)	28 (20.7%)	
No	35 (83.3%)	107 (79.3%)	
T Category			0.751
1a	37 (88.1%)	121 (89.6%)	
1b	2 (4.8%)	8 (5.9%)	
3b	3 (7.1%)	6 (4.4%)	
N Category			0.414
0	32 (76.2%)	94 (69.6%)	
1a	10 (23.8%)	41 (30.4%)	
Stage			0.843
I	41 (97.2%)	131 (97%)	
II	1 (2.8%)	4 (3%)	

Values are numbers of patients(percentages) unless otherwise indicated

*Abbreviations*: *PTC* papillary thyroid carcinoma, *FTC* Follicular thyroid carcinoma, *LN* lymph node, *TT* total thyroidectomy with CCND, *LT* Lobectomy with CCND, *BMI* Body Mass Index

### Surgical outcomes

Table 3 shows the surgical outcomes of 177 patients after propensity score matching. The mean operation time was significantly longer in the EG than in the RG (207.5 ± 50.8 min in EG vs. 132.9 ± 43.1 min in RG, *p* < 0.001). Furthermore, more retrieved central lymph nodes were removed by RG (5.4 ± 3.6 in EG vs. 11.9 ± 6.1 in RG, *p* < 0.001). Besides, the mean maximum diameter of the thyroid was more expansive in the EG than in the RG (5.15 ± 0.90 cm in EG vs. 4.93 ± 0.91 cm in RG, *p* = 0.04). There were no significant differences in the total amount of drainage and drain insertion between the two groups (239.8 ± 100.0 ml in EG vs. 266.0 ± 150.2 ml in RG, *p* = 0.241 and 5.79 ± 1.6 day in EG vs. 6.15 ± 2.04 day in RG *p* = 0.316, respectively). Both groups showed that cosmetic satisfaction (*p* = 0.837) and postoperative hospitalized stay (*p* = 0.100) were similar. Furthermore, positive LN metastasis (*p* =0.561) and pain score (*p* = 0.077) showed no significant difference between the two groups.

**Table 3:** Postoperative outcomes in the robotic and endoscopic groups

	**Endoscopic Thyroidectomy (*N*** = **42)**	**Robot-assisted Thyroidectomy (*N*** = **135)**	***P***
Total operation time(min)	207.5 ± 50.8	132.9 ± 43.1	≤0.001
Total amount of drainage (ml)	239.8 ± 100.0	266.0 ± 150.2	0.241
Drain insertion (days)	5.79 ± 1.60	6.15 ± 2.04	0.316
Maximum diameter of thyroid (cm)	5.15 ± 0.90	4.93 ± 0.91	0.040
Positive LN^a^ metastasis (n)	3 ± 2.31	2.3 ± 1.73	0.561
Retrieved central LN^a^(n)	5.4 ± 3.6	11.9 ± 6.1	≤0.001
Cosmetic satisfaction^b^			0.837
3	34 (81.0%)	111 (82.2%)	
2	6 (14.3%)	19 (14.1%)	
1	2 (4.8%)	5 (3.7%)	
Pain score (VAS)^c^	1.42 ± 0.74	1.34 ± 0.94	0.077
Post operative hosipitial stay(d)	7.19 ± 1.60	7.92 ± 2.34	0.100

Value are means ± SD

The underlined values in the table show statistically significant difference between the two groups

^a^*LN* lymph node

^b^Extremely satisfied (3point), satisfied (2 point), acceptable (1 point), and dissatisfied (0point)

^c^VAS ranges from 0 (no pain) to 10 (worst pain). *VAS* Visual Analogue Scale

### Relationship between operation time and number of cases

**Fig. 3:**
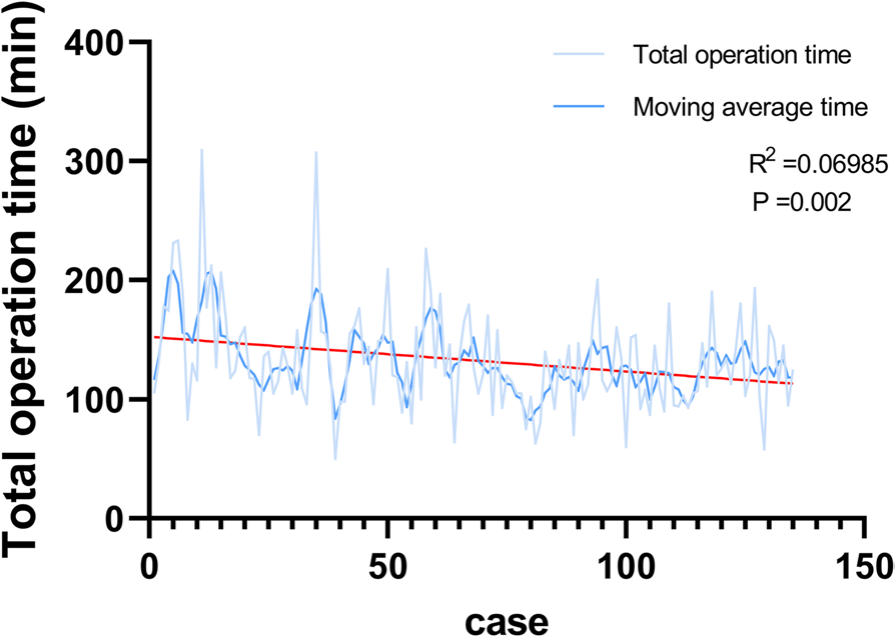
The operative times of the 135 consecutive patients who underwent robotic thyroidectomy using the bilateral axillary breast approach (BABA)

The relationship between the operation time and the number of operations is shown in Fig. 3. One hundred thirty-five patients underwent robotic-assisted thyroidectomy in this study. Using the simple linear regression model and smooth curve (moving average time), the operation times for RG trended to decrease gradually as the cases went by. (95% CI -0.4735 to -0.1090, *P*= 0.002).

### Complications

Table 4 shows the two groups’ postoperative complications and postoperative laboratory data. The mean postoperative PTH and total calcium were nonsignificantly between the 2 groups (21.1± 12.4 pg/mL in EG vs 20.7 ± 15.1 pg/mL in RG *P*=0.659, 2.19 ± 0.14 ng/mL in EG vs2.14 ± 0.13 ng/mL in RG *P*=0.068, respectively). The two groups had the same rate of hypoparathyroidism during the surgery (*p* = 0.331). There was less hypocalcemia in the EG, but there was no significant difference between the two groups (19 % in ET vs. 34.1 % in RT, *p* = 0.065). Only one patient in the RG developed seroma (0.7%). Besides, two patients developed chyle leakage (1.5%), but both groups observed no significant infection, bleeding, RLN injury or flap necrosis during hospitalization.

**Table 4:** Postoperative laboratory data and complications of patients in the robotic and endoscopic groups

	**Endoscopic Thyroidectomy (*N*** = **42)**	**Robot-assisted Thyroidectomy (*N*** = **135)**	***P***
Postoperative total calcium (ng/mL)	2.19 ± 0.14	2.14 ± 0.13	0.068
Postoperative PTH^a^ (pg/mL)	21.1 ± 12.4	20.7 ± 15.1	0.659
Hypocalcemia(n)^b^			
Transient	8 (19.0%)	46 (34.1%)	0.065
Permanen	0	0	-
Hypoparathyroidism(n)^c^			
Transient	19 (74.8%)	17 (74.8%)	0.331
Permanen	0	0	-
Infection	0	0	-
Bleeding	0	0	-
Chyle leakage	0	2 (1.5%)	1.0^d^
RLN injury	0	0	-
Flap necrosis	0	0	-
Seroma	0	1 (0.7%)	1.0^d^

Value are means ± SD

Values are numbers of patients(percentages) unless otherwise indicated

^a^PTH parathyroid hormone

^b^Hypocalcemia: calcium < 2.08 ng/mL or symptom ( +) after total thyroidectomy

^c^Hypoparathyroidism: parathyroid hormone < 15.0 pg/mL after total thyroidectomy

^d^Fisher’s exact test

## Discussion

In our present study, we provide evidence that robotic thyroidectomy offers many potential advantages over endoscopic thyroidectomy, including retrieving more central lymph nodes and shortening the procedure time. Furthermore, the procedure times for RG decreased gradually as the cases went by. However, the larger diameter of the thyroid specimen was removed by endoscopic. To clarify the advantages of these two surgical procedures, PSM analysis on the baseline data was performed to minimize biases in compared 2 Groups. Then, we compared the two groups postoperative outcomes and histopathologic characteristics. Our results showed that robotic-assisted surgical procedures have advantages over endoscopic lymph node retrieval and operative time. In contrast, endoscopic thyroidectomy procedures have broader applicability than robotic-assisted surgical procedures for the larger diameter of thyroid specimens.

With the innovation of robot thyroidectomy, RT using the same approaches as ET is widely developed [21]. Robotic thyroidectomy using the BABA approach was first reported by Choe [22]. According to many previous studies on patients undergoing endoscopic and robot thyroidectomy, there was a significant difference in the level of the operation time, number of retrieved central LN, and cosmetic satisfaction compared with conventional open thyroidectomy [14, 23]. There have been some studies on this remote access that compared the postoperative pathological and surgical outcomes including complications between endoscopes and robotics, and these studies found that the robot has many advantages over the endoscopic thyroidectomy, such as providing a 3-D view of the surgery, the robot’s robotic arm removes tremors, and the three robotic arms allow for more delicate anatomy of the operative area [12–14, 24, 25]. To our knowledge, there was no report of the comparison of BAA in endoscopes and BABA in robotics. Therefore, we choose the endoscopic BAA approach and the robotic BABA approach to study.

The superiority of robots in operation time has been demonstrated in two ways. On the one hand, we found that the mean operation time of EG was significantly longer than RG (Table 3). This proves that robotic-assisted surgical procedures take less time to complete the procedures. In contrast, others have shown that the robot thyroidectomy procedure takes more time than the endoscopic [13, 14, 26]. This may be due to our extensive experience in robotic surgery, and we have less docking time.

Furthermore, like the endoscopic approach, we still have an assistant to help the instrument nurse change instruments, wipe the cavity lens, and take out the specimen. Repeated connection and disconnection from robotic arms could be a time-consuming procedure for robotic surgery compared to the endoscopic approach [14]. These difficulties can be overcome with the cooperation of a trained surgical assistant. In addition, previous studies have focused on a single contrasting axillary approach, transoral approach, or BABA approach, respectively [12, 25]. As reported in a study by Kim [9], the BAA procedure requires longer operating times than the BABA procedure. The longer operating times were probably because of more difficulty handling the operation instruments. So the total surgery time may be related to our chosen remote access approaches.

On the other hand, the simple linear regression between the operation time and surgery cases shows that the operation times for RG decreased gradually as the cases went by (Fig. 3). In contrast, there was no statistical difference between surgery cases and operation time in endoscopic surgery. This indicates greater surgical efficiency with accumulated experience and cases in RT. Meanwhile, the RG had retrieved more central lymph nodes than EG, indicating that robots are superior in the dissection of lymph nodes than endoscopy. This may be attributed to the advantages of the robot procedure in three-dimensional magnification and precise manipulation of instruments without tremor [12, 13, 25]. Similar observations have been documented for the significant difference in the number of retrieved central lymph nodes between endoscopic and robotic group [12]. Likewise, they found that the robot procedure has an advantage over lymph node dissection compared with the endoscopic procedure. This means that robot procedure has a wider extent and radical operations. As reported in a study by Lee, robotic thyroidectomy was more advanced than endoscopic thyroidectomy in terms of operative time, lymph node dissection, and learning curve [27]. This significant difference in the number of CLNs resected may be meaningful for DTC patients who will receive therapeutic CND [28, 29].

However, the mean maximum diameter of thyroid specimens was more expansive in the EG than in the RG (Table 3), which also provided evidence of a more radical approach in EG to surgical indications. We did not include the thyroid gland size in the covariates because the size of the thyroid gland of the included patients did not affect the surgical approach. The endoscope can use the human hand’s flexibility without considering the distance between the robotic arms to make a larger angle than the robot. This may be the reason why endoscopic thyroidectomy can remove larger thyroid glands.

Due to men’s more prominent musculoskeletal structure, which poses greater technical challenges than women’s [14], the proportion of male patients who chose RT was much higher than the ET group. The superiority of the robot in removing lymph nodes may have led to a preference for robotic surgery in more PCT patients [27, 30]. In addition, according to the ACJJ [31], the category of T1a patients more frequently underwent robotic thyroidectomy than endoscopic. In comparison, more categories of T1b and T3b underwent endoscopic thyroidectomy.

Unlike our study, a previous trial showed that RT had more frequent hypocalcemia. They think it might be due to the complete cleaning of thyroid tissue. Complete perithyroidal fascia and soft tissue removal may lead to transient thermal damage to the parathyroid gland or transient ischemia [12]. Through our research, we have shown that through the increase in surgical proficiency, both lymph node removal and prevention of postoperative complications can be taken into account. At the same time, special care should be taken to protect the parathyroid glands while performing surgery. As reported in previous studies [32], in more invasive surgery, the harmonic scalpel and hemostatic powder are required to minimize the risk of Hemostasis and complications during thyroidectomy. In our study, hypoparathyroidism, hypocalcemia, infection, bleeding, flap necrosis, and RLN injury were absent in either group of procedures. This is consistent with the results of a previous meta-analysis [33]. Past studies found that the completeness of robotic lymph node dissection may impact postoperative drainage [13]. However, this study did not find a significant difference in the total amount of drainage, drain insertion (days), and postoperative hospital stay. No significant differences were found in cosmetic satisfaction. A similar report has demonstrated that ET and RT both have superior cosmetic effects to conventional open surgery [12].

Since our department completed the first da Vinci robot-assisted thyroidectomy in mainland China in 2014, we have had more than 2 thousand successful cases of robotic surgery. The major strength of this study was that we accumulated a large amount of clinical data and surgical experience to support this study. As we know, we first showed the comparison of the robotic BABA approach and the endoscopic BAA approach using propensity score matching. Furthermore, our findings may carry more credibility than other studies because all comparisons, including surgical outcome and postoperative pathology, were performed under strictly matched conditions.

However, as this study is retrospective, the main limitation of this study is that selection bias cannot be eliminated, and the unobserved covariate values cannot be balanced by propensity score matching. So this study cannot replace the randomization process. The comparison of these two groups requires further prospective controlled studies. Furthermore, complications and recurrence need to be further studied in long-term follow-up. In addition, although we used a 1:4 ratio of matching to balance the two groups, too few endoscopic cases were included in this study. Including patients who underwent thyroidectomy with lateral neck dissection for DTC, long-term follow-up results, and other approaches in robotic or endoscopic thyroidectomy are required in further studies.

## Conclusion

For patients who underwent CND, the two thyroidectomies have the same treatment effect on DTC. As this study shows, the advantages of robotic thyroidectomy include shorter operating times and complete lymph node removal. With the number of robotic surgical cases increasing, the operation time is accelerated leading to higher operational efficiency. In contrast, endoscopic thyroidectomy has lower surgical costs due to less dependence on surgical equipment. Besides, it resects a larger thyroid and has fewer postoperative complications than robotic thyroidectomy. In conclusion, robotic and endoscopic thyroidectomy are similar minimally invasive thyroid surgeries, each with its advantages, both of which can achieve the expected surgical outcomes.

### Supplementary Information

**Additional file 1.**

## Data Availability

The datasets used and/or analyzed during the current study are available from the corresponding author (Luming Zhen) upon reasonable request. For any queries, kindly contact luming720513@163.com.
